# Malignant melanoma is genetically distinct from clear cell sarcoma of tendons and aponeurosis (malignant melanoma of soft parts)

**DOI:** 10.1054/bjoc.2000.1628

**Published:** 2001-02

**Authors:** S M Langezaal, J F Graadt van Roggen, A M Cleton-Jansen, J J Baelde, P C W Hogendoorn

**Affiliations:** Department of Pathology, Leiden University Medical Center, Leiden, The Netherlands

**Keywords:** soft tissue tumour, sarcoma, clear cell sarcoma, malignant melanoma of soft parts, melanoma, chromosomal rearrangement; RT-PCR

## Abstract

Clear cell sarcoma of tendons and aponeuroses (malignant melanoma of soft parts) and conventional malignant melanoma may demonstrate significant morphologic overlap at the light microscopic and ultrastructural level. Consequently, the clinically relevant distinction between primary clear cell sarcoma and metastatic melanoma in the absence of a known primary cutaneous, mucosal or ocular tumour may occasionally cause diagnostic problems. A balanced translocation, t(12;22)(q13;q13), which can be detected, amongst others, using the reverse transcriptase polymerase chain reaction (RT-PCR) or fluorescent in situ hybridization (FISH), has been identified in a high percentage (50–75%) of clear cell sarcomas and is presumed to be tumour specific. Whether this chromosomal rearrangement is present in malignant melanoma has, to date, not as yet been studied by molecular genetic or molecular cytogenetic techniques. Using RT-PCR and FISH, a series of metastases from 25 known cutaneous melanomas and 8 melanoma cell lines (5 uveal and 3 cutaneous) were screened for the t(12;22)(q13;q13) translocation. Primers for RT-PCR were chosen based upon published breakpoint sequences. The Cosmids G9 and CCS2.2, corresponding to the 5′ region of EWS and 3′ region of ATF-1 respectively, were used as probes. The translocation was not identified in any of the melanomas or melanoma cell lines analysed in this study; in contrast this translocation was identified in 3 out of 5 clear cell sarcomas using these techniques. This allows distinction between translocation positive cases of clear cell sarcoma and malignant melanoma at a molecular genetic level. Consequently, in diagnostically challenging cases, this represents a valuable tool for the clinicopathologic differentiation between these two entities, with an important impact on patient management and prognosis. © 2001 Cancer Research Campaign http://www.bjcancer.com


British Journal of Cancer (2001) 84(4), 535–538
© 2001 Cancer Research Campaign
doi: 10.1054/ bjoc.2000.1628, available online at http://www.idealibrary.com on IDEAL®

### Malignant melanoma is genetically distinct from clear cell sarcoma of tendons and aponeurosis (malignant melanoma of soft parts)

**SM Langezaal, JF Graadt van Roggen, AM Cleton-Jansen, JJ Baelde and PCW Hogendoorn**

Department of Pathology, Leiden University Medical Center, Leiden, The Netherlands

**Summary** Clear cell sarcoma of tendons and aponeuroses (malignant melanoma of soft parts) and conventional malignant melanoma may demonstrate significant morphologic overlap at the light microscopic and ultrastructural level. Consequently, the clinically relevant distinction between primary clear cell sarcoma and metastatic melanoma in the absence of a known primary cutaneous, mucosal or ocular tumour may occasionally cause diagnostic problems. A balanced translocation, t(12;22)(q13;q13), which can be detected, amongst others, using the reverse transcriptase polymerase chain reaction (RT-PCR) or fluorescent in situ hybridization (FISH), has been identified in a high percentage (50–75%) of clear cell sarcomas and is presumed to be tumour specific. Whether this chromosomal rearrangement is present in malignant melanoma has, to date, not as yet been studied by molecular genetic or molecular cytogenetic techniques. Using RT-PCR and FISH, a series of metastases from 25 known cutaneous melanomas and 8 melanoma cell lines (5 uveal and 3 cutaneous) were screened for the t(12;22)(q13;q13) translocation. Primers for RT-PCR were chosen based upon published breakpoint sequences. The Cosmids G9 and CCS2.2, corresponding to the 5′ region of EWS and 3′ region of ATF-1 respectively, were used as probes. The translocation was not identified in any of the melanomas or melanoma cell lines analysed in this study; in contrast this translocation was identified in 3 out of 5 clear cell sarcomas using these techniques. This allows distinction between translocation positive cases of clear cell sarcoma and malignant melanoma at a molecular genetic level. Consequently, in diagnostically challenging cases, this represents a valuable tool for the clinicopathologic differentiation between these two entities, with an important impact on patient management and prognosis. © 2001 Cancer Research Campaign http://www.bjcancer.com

**Keywords**: soft tissue tumour; sarcoma; clear cell sarcoma; malignant melanoma of soft parts; melanoma; chromosomal rearrangement; RT-PCR

Clear cell sarcoma of tendons and aponeuroses (CCS), also known as malignant melanoma of soft parts, represents a distinct clinicopathologic entity first described by Enzinger in 1965 (Enzinger, 1965). CCS usually presents as a slowly growing and frequently painful soft tissue mass, occuring primarily in adolescents and young adults, with a predilection for the lower extremeties, especially around the ankle and knee, although the overall anatomic distribution is wide (Chung and Enzinger, 1983). The tumours are usually intimately associated with fascial or tendoaponeurotic structures but may regularly involve the subcutis and dermis by direct extension. Radiologically the lesion is often mistaken for a benign process (De Beuckeleer et al, 2000). CCS is characterized by repeated local recurrence and, with time, metastasizes primarily to lymph nodes, the lungs and bone; 5 and 10 year survival is variable but is in the order of 50% and 10–20%, respectively. Surgery remains the mainstay of therapy while the efficacy of adjuvant radiotherapy and/or chemotherapy remains uncertain.

Histopathologically, CCS displays a number of features exhibiting a broad morphologic overlap with conventional malignant melanoma, including melanin synthesis and expression of HMB-45 and S-100 protein (Kindblom et al, 1983; Mooi et al, 1995; Graadt van Roggen et al, 1998). In addition vimentin and, occasionally, low levels of low molecular weight cytokeratins are also variably present (Mooi et al, 1995). Ultrastructurally, features of melanocytic differentiation are identifiable in both tumours including the presence of an external lamina and cytoplasmic premelanosomes in various stages of maturation (Kindblom et al, 1983).

Cytogenetic analysis of CCS has identified the presence of an apparently tumour-specific balanced translocation involving chromosomes 12 and 22 in more than 50% of cases (Epstein et al, 1984; Bridge et al, 1990, 1991; Peulvé et al, 1991; Fletcher, 1992; Reeves et al, 1992; Rodriguez et al, 1992; Speleman et al, 1992; Stenman et al, 1992; Travis and Bridge, 1992; Mrozek et al, 1993; Limon et al, 1994; Zucman et al, 1993; Nedoszytko et al, 1996; Speleman et al, 1997; Graadt van Roggen et al, 1998). This translocation results in the fusion of a portion of the Activating Transcription Factor gene (*ATF-1*) on the long arm of chromosome 12(12q13.1–13.2) and the Ewing's sarcoma oncogene (*EWS*) on chromosome 22 (22q13) (Zucman et al, 1993). To date no significant study has investigated the presence or absence of this translocation in malignant melanoma in order to validate the value of detection of this translocation in distinguishing between translocation-positive CCS and metastatic melanoma in the absence of a known primary tumour. Since the distinction between these two entities may be of significant clinical importance, and since histomorphologic examination may not always provide an unambiguous diagnosis, the presence of a

*Received 16 August 2000*
*Revised 6 November 2000*
*Accepted 8 November 2000*

*Correspondence to:* PCW Hogendoorn, Leiden University Medical Center, Dept of Pathology, PO Box 9600, L19, 2300 RC, Leiden, The Netherlands. E-mail: P.C.W.Hogendoorn@lvmc.nl

(cyto)genetically detectable molecular genetic alteration to make this distinction is very attractive.

For this reason metastases of 25 primary cutaneous melanomas as well as 8 well characterized melanoma cell lines (5 uveal and 3 ocular) were analysed for the possible presence of the t(12;22)(q13;q13) translocation using the reverse transcriptase polymerase chain reaction (RT-PCR) and interphase fluorescent in situ hybridization (FISH) using chromosome 12 (*ATF-1*) c.q. chromosome 22 (*EWS*) specific probes.

### MATERIALS AND METHODS

### Materials

Snap frozen tissue samples stored at –80˚C, of melanoma metastases from 25 patients with a known cutaneous malignant melanoma, were retrieved from our archives. Histologic slides of both the primary tumours and metastases were independently reviewed by two pathologists (JFGvR and PCWH). Furthermore, we obtained RNA from 5 uveal and 3 cutaneous melanoma cell lines kindly provided by Dr Martine J Jager (de Waard-Siebinga et al, 1995). As a positive control for the RT-PCR and FISH studies we used the SU-CCS-1 clear cell sarcoma cell line, cytogenetically proven to carry the t(12;22)(q13;q13) translocation, generously provided by Dr O Delattre (Zucman et al, 1993). Snap frozen tissue from 2 cases of CSS in our archives carrying the t(12;22)(q13;q13) translocation as detected using RT-PCR (see below) were used as additional positive controls. MDA-MB-134, a breast cancer cell line was used as a negative control.

### Methods

#### *RNA and DNA analysis*

Total RNA was extracted from the various cell lines ($4.5 \times 10^6$ cells per extraction) using TRIzol (Gibco BRL Life Technologies, Gaithersburg, MD, USA), according to the manufacturer's recommendation. RNA from snap frozen tumour tissue was isolated using TRIzol after cutting 20 sections of 20 µm on a cryostat-microtome. Between cutting of individual specimens, the microtome blade was thoroughly cleaned with 70% ethanol to avoid cross contamination. The RNA concentration and purity was determined by measuring the absorption at 260 and 280 nm spectrophotometrically. cDNA was made from total RNA using AMV-reverse transcriptase (Boehringer Mannheim, Germany). One to two µg of total RNA was used for reverse transcription. The RNA was added to a mixture of 100 ng 15-mer primer pDT (Boehringer Mannheim, Germany), 1U RNAsin, 1mM dNTP, 100 mM Tris-HCL pH of 8.3, 80 mM KCL, 12 mM $MgCl_2$, 2 mM DTT and 5 U AMV-reverse transcriptase up to a volume of 20 µl. The tubes were incubated at 39˚C for one hour. The tubes, now containing cDNA, were stored at –20˚C or used directly. One µl of the cDNA was used for amplification. The cDNA was added to a mixture containing 30 pmol of a forward primer in exon 8 of EWS designated as DO3 (5′-ATCGTGGAGGCATGAGCA-3′) and a reverse primer in ATF-1 designated as DO4 (5′-ACTCCATCTGTGC-CTGGACT-3′) (Life Technologies, Gaithersburg, MD, USA), 0.04 mM dNTP, 50 mM KCl, 10 mM Tris (pH 8.3), 0.2 mg $ml^{-1}$ bovine serum albumin (BSA), 2 mM $MgCl_2$ and 1 U AmpliTaqGold (PE Biosystems) up to a volume of 50 µl. Subsequently PCR-reactions were performed using conditions deduced from a Robocycler gradient 96 experiment (Stratagene). Optimal PCR conditions were 33 cycles, with each cycle consisting of denaturation at 94˚C for 30 sec, annealing at 65˚C for 1 min and elongation at 72˚C for 1 min. Optimal $MgCl_2$ concentration appeared to be 2 mM. Transcripts of the housekeeping gene for hypoxanthine phosphoribosyl transferase (HPRT) were amplified simultaneously under the same conditions as a control for RNA integrity. HPRT cDNA was amplified with primers hum 1 (5′-ACCG-GCTTCCTCCTCCTGAGCAGTC-3′) and hum2 (5′-AGGACTC-CAGATGTTTCCAAACTCAACTT-3′). cDNA from a breast cancer cell line (MDA-MB-134) was used as a negative control for the RT-PCR translocation. cDNA from the cultured CCS cell line, with cytogenetically confirmed t(12;22)(q13;q13), was included in each test as a positive control for the EWS/ATF-1 fusion transcript. Water was used as a negative control to exclude the possibility of contamination of reagents. Thermal cycling was performed in a DNA Thermal cycler Type 480, Perkin Elmer.

RT-PCR products were stained with ethidium bromide and visualized on a 1.5% agarose gel. Sequence analysis was performed on an AB1 377 automatic sequencer using a Big Dyeterminator sequencing kit (PE Biosystems).

#### *Fluorescent in situ hybridization*

Interphase FISH was performed on nuclei as described (Vaandrager et al, 1996). The nuclei were isolated from sections of frozen tissue of 40 µm. Nuclei isolated from the SU-CCS-1 cell line, with a proven t(12;22) translocation, were used as a positive control.

The Cosmids G9 and CCS2.2 (generously provided by Dr Speleman) (Speleman et al, 1997), corresponding to the 5′region of EWS and 3′ region of ATF-1 respectively, were used as probes and labelled by a standard nick-translation method. Cosmid G9 was labelled with biotin-16-dUTP and CCS2.2 with digoxine-11-dUTP (Roche, Basle, Switzerland). Slides were hybridized overnight with hybridization solution containing 50% formamide, 10% dextran sulphate, 50 mM sodium phosphate, 3 ng $\mu l^{-1}$ of each probe and a 50-fold excess of human Cot-1 DNA. Hybridization and immunofluorescence detection were performed as described (Vaandrager et al, 1996). 100 nuclei of each hybridization were analysed for colocalization of both signals. The t(12;22) translocation was diagnosed when colocalization of signal was seen in more than 10% of the nuclei studied.

### RESULTS

None of the 25 melanoma metastases and 8 melanoma cell lines carried the t(12;22)(q13;q13) translocation as analysed by RT- PCR. The SU-CCS-1 cell line with a cytogenetically proven t(12;22)(q13;q13) translocation, as well as two clear cell sarcomas, demonstrated the EWS/ATF-1 fusion transript using RT- PCR.

The EWS/ATF-1 fusion transcript in all positive control cases studied yields a 241 base pair (bp) PCR product (Figure 1), as expected from previous studies (Zucman et al, 1993). This is the result of a fusion between codon 325 of the EWS gene and codon 65 of ATF-1. The chimaeric breakpoint was confirmed by sequence analysis of the RT-PCR product (data not shown). RNA integrity of all negative cases was assessed by RT-PCR amplification of the housekeeping gene HPRT, which generates a 747 bp PCR product. In all tumours and cell lines tested HPRT was clearly and unambiguously demonstrable.

 © 2001 Cancer Research Campaign

FISH on interphase nuclei was performed to confirm the RT-PCR results (Figure 2). None of the melanomas tested carried the t(12;22)(q13;q13) translocation not detectable using RT-PCR, while the SU-CCS-1 cell line and the two CCS with the, by RT-PCR detectable, t(12;22)(q13;q13) translocation showed colocalization of the EWS and ATF probes.

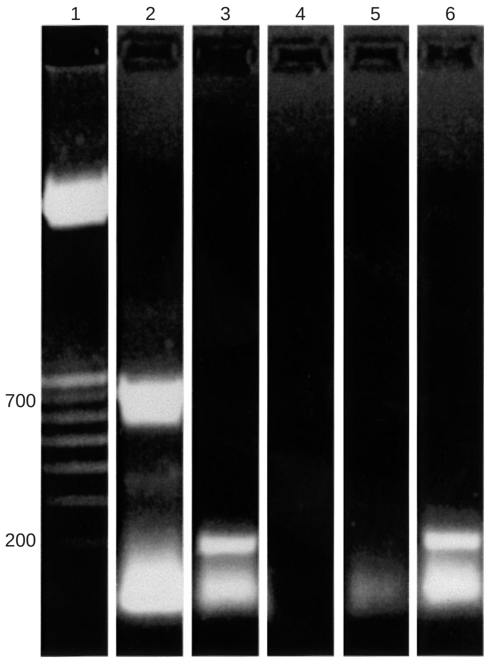

**Figure 1** Reverse transcriptase polymerase chain reaction (RT-PCR) assay used to screen the melanoma metastases and melanoma cell lines for the t(12;22) translocation. Following RT-PCR, the reaction products were run on a 1.5% agarose gel and visualized using ethidium bromide. Lane 1: 100 bp marker ladder, lane 2: HPRT amplification to control for RT-PCR reaction, lane 3: SU-CCS-1 positive control cell line carrying t(12;22) translocation, lane 4: 'no template' negative control, lane 5: melanoma metastasis, lane 6: clear cell sarcoma

### DISCUSSION

In the last decade it has become increasingly apparent that specific soft tissue tumours may be associated with tumour-specific translocations (Fletcher et al, 1991; Ladanyi, 1995; Graadt van Roggen et al, 1999). Tumour-specific (cyto)genetic alterations may be particularly useful in the diagnostic setting where the histopathologic features and conventional ancillary techniques are insufficient to allow an unambiguous diagnosis (e.g. in the differential diagnosis of small round blue cell tumours), and where an accurate histopathologic diagnosis will significantly influence the therapeutic modalities employed. In the appropriate clinico-pathologic setting the relevant differential diagnosis with CCS and metastatic malignant melanoma without a known primary may occasionally be problematic due to the broad morphologic overlap which exists between these two tumours. Nevertheless, an accurate histopathologic diagnosis is of paramount importance considering the different therapeutic options which may be applicable in the individual clinical setting when considering a primary soft tissue tumour versus a metastatic process.

In CCS a t(12;22)(q13;q13) translocation has been characterized and appears to be specific for this sarcoma (Zucman et al, 1993; Ladanyi, 1995; Speleman et al, 1997; Graadt van Roggen et al, 1999). Cytogenetic studies of melanomas, while identifying numerous non-specific and variable chromosomal alterations, have not as yet identified the t(12;22) translocation within this tumour (Pedersen et al, 1985; Graadt van Roggen et al, 1998; Piepkorn, 2000). At a molecular level however, data is beginning to support the probable involvement of the CDKN2A tumour-suppressor locus and other loci in tumorigenesis (Castellanol and Parmiani, 1999; Piepkorn, 2000).

Consequently, detection of the t(12;22) translocation by molecular biological techniques (eg. RT-PCR or FISH) may be very useful in distinguishing CCS and metastatic melanoma in the appropriate clinical setting.

Two variant hybrid t(12;22) transcripts have been identified. The breakpoints differ by 40 amino acids, but since both chimaeras contain essentially the same regions the biological behaviour of these molecular variants is not expected to be dissimilar (Speleman et al, 1997; Graadt van Roggen et al, 1998).

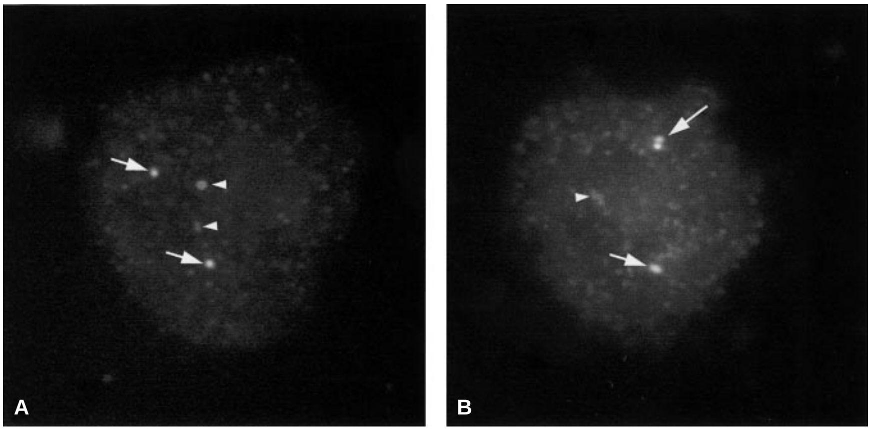

**Figure 2** Fluorescent in situ hybridization performed to validate the reverse transcriptase polymerase chain reaction results described under 'Results' Arrowheads represent *EWS* probe on chromosome 22; short-tailed arrows represent *ATF-1* probe on chromosome 12. **(A)** Two copies of both chromosomes 12 and 22 are visualized using the respective probes for *ATF-1* and *EWS* (see methods). **(B)** Colocalization (long-tailed arrow) of both probes confirming the presence of the t(12; 22) translocation in the SU-CCS-1 cell line used as positive control

© 2001 Cancer Research Campaign

The first hybrid consists of EWS [exon 8, (codon 325)/ATF-1 (codon 65)], which gives a PCR-fragment of 241 bp using our primer constructs. The second hybrid consist of EWS [exon 10, 11 junction (codon 349)/ATF-1 (codon 110)], which would give a PCR-fragment of 178 bp with our primer constructs (Zucman et al, 1993; Speleman et al, 1997; Graadt van Roggen et al, 1998).

To date no systematic investigations of the presence or absence of this translocation in malignant melanoma have been published, which was the main motive for initiating this analysis. Our results clearly demonstrate the absence of the t(12;22)(q13;q13) in any of the melanoma samples tested, in the presence of positive and negative controls. Although RT-PCR is sensitive and reliable, there is a possibility that additional, as yet undescribed, variant EWS/ATF-1 translocations might be missed if the chromosomal breakpoints lie outside the primer set used in this study. Consequently FISH studies were performed, as a means of verifying the negative RT-PCR results, and to identify any possible additional chromosomal EWS/ATF-1 rearrangements. The FISH results however confirmed our RT-PCR data and failed to identify any additional translocations involving EWS and ATF-1.

In CCS as well as the SU-CCS-1 cell line the t(12;22) translocation was clearly detected with RT-PCR, as well as FISH.

Despite the morphologic similarity between CCS and metastatic melanoma at a light microscopic and ultrastructural level, clearly these tumours are distinct entities at the molecular genetic level supporting the supposition that CCS represents a separate and distinct entity. Nevertheless, the t(12;22)(q13;q13) translocation only appears to be detectable in 50–70% of cases of clear cell sarcoma. Therefore the absence of a detectable EWS/ATF-1 rearrangement may on occasion lead to the erroneous exclusion of a translocation negative CCS in the above mentioned clinicopathological differential diagnosis. This needs to be borne in mind when the discussed differential diagnosis between clear cell sarcoma and metastatic melanoma arises and resolution will require review of the case in the context of the complete clinicopathologic setting.

### REFERENCES

Bridge JA, Borek DA, Neff JR and Huntrakoon M (1990) Chromosomal abnormalities in clear cell sarcoma. Implications for histogenesis. *Am J Clin Pathol* **93**: 26–31

Bridge JA, Sreekantaiah C, Neff JR and Sandberg AA (1991) Cytogenetic findings in clear cell sarcoma of tendons and aponeuroses. Malignant melanoma of soft parts. *Cancer Genet Cytogenet* **52**: 101–106

Castellano M and Parmiani G (1999) Genes involved in melanoma: an overview of INK4a and other loci. *Melanoma Res* **9**: 421–432

Chung EB and Enzinger FM (1983) Malignant melanoma of soft parts. A reassessment of clear cell sarcoma. *Am J Surg Pathol* **7**: 405–413

De Beuckeleer LH, De Schepper AM, Vandevenne JE, Bloem JL, Davies AM, Oudkerk M, Hauben E, Van Marck E, Somville J, Vanel D et al (2000) MR imaging of clear cell sarcoma (malignant melanoma of the soft parts): a multicenter correlative MR-pathology study of 21 cases and literature review. *Skelet Radiol* **29**: 187–195

de Waard-Siebinga I, Blom DR, Griffioen M, Schrier PI, Hoogendoorn E, Beverstock GC, Danen EHJ, and Jager MJ (1995) Establishment and characterization of an uveal-melanoma cell line. *Int J Cancer* **62**: 155–161

Enzinger FM (1965) Clear-cell sarcoma of tendons and aponeuroses. An analysis of 21 cases. *Cancer* **18**: 1163–1174

Epstein IL, Martin AO and Kempson R (1984) Use of a newly established cell line (SU-CCS-1) to demonstrate the relationship of clear cell sarcoma to malignant melanoma. *Cancer Res* **44**: 1265–1274

Fletcher JA (1992) Translocation (12;22)(q13–14;q12) is a nonrandom aberration in soft-tissue clear-cell sarcoma. *Genes Chrom Cancer* **5**: 184

Fletcher JA, Kozakewich HP, Hoffer FA, Lage JM, Weidner N, Tepper R, Pinkus GS, Morton CC and Corson JM (1991) Diagnostic relevance of clonal cytogenetic aberrations in malignant soft-tissue tumors. *N Engl J Med* **324**: 436–442

Graadt van Roggen JF, Mooi WJ and Hogendoorn PCW (1998) Clear cell sarcoma of tendons and aponeuroses (malignant melanoma of soft parts) and cutaneous melanoma: exploring the histogenetic relationship between these two clinicopathological entities. *J Pathol* **186**: 3–7

Graadt van Roggen JF, Bovee JVMG, Morreau J and Hogendoorn PCW (1999) Diagnostic and prognostic implications of the unfolding molecular biology of bone and soft tissue tumours. *J Clin Pathol* **52**: 481–489

Kindblom L-G, Lodding P and Angervall L (1983) Clear-cell sarcoma of tendons and aponeuroses. An immunohistochemical and electron microscopic analysis indicating neural crest origin. *Virchows Arch* **401**(A): 109–128

Ladanyi M (1995) The emerging molecular genetics of sarcoma translocations. *Diagn Mol Pathol* **4**: 162–173

Limon J, Debiec-Rychter M, Nedoszytko B, Liberski P, Babinska M and Szadowska A (1994) Aberrations of chromosome 22 and polysomy of chromosome 8 as non-random changes in clear cell sarcoma. *Cancer Genet Cytogenet* **72**: 141–145

Mooi WJ, Deenik W, Peterse JL and Hogendoorn PCW (1995) Keratin immunoreactivity in melanoma of soft parts (clear cell sarcoma). *Histopathology* **27**: 61–65

Mrozek K, Karakousis CP, Perez-Mesa C and Bloomfield CD (1993) Translocation t(12;22)(q13;q12.2-12.3) in clear cell sarcoma of tendons and aponeuroses. *Genes Chrom Cancer* **6**: 249–252

Nedoszytko B, Mrozek K, Roszkiewicz A, Kopacz A, Swierblewski M and Limon J (1996) Clear cell sarcoma of tendons and aponeuroses with t(12;22)(q13;q12) diagnosed initially as malignant melanoma. *Cancer Genet Cytogenet* **91**: 37–39

Pedersen MI, Bennett JW and Wang N (1985) Nonrandom chromosomes, structural aberrations, and oncogenic loci in human malignant melanoma. *Cancer Genet Cytogenet* **20**: 11–27

Peulvé P, Michot C, Vannier JP, Tron P and Hemet J (1991) Clear cell sarcoma with t(12;22)(q13-14;q12). *Genes Chromosomes Cancer* **3**: 400–402

Piepkorn M (2000) Melanoma genetics: an update with focus on the CDKN2A(p16)/ARF tumor suppressors. *J Am Acad Dermatol* **42**: 705–722

Reeves BR, Fletcher CDM and Gusterson BA (1992) Translocation t(12;22)(q13;q13) is a nonrandom rearrangement in clear cell sarcoma. *Cancer Genet Cytogenet* **64**: 101–103

Rodriguez E, Sreekantaiah C, Reuter VE, Motzer RJ and Chaganti RSK (1992) t(12;22)(q13;q13) and trisomy 8 are nonrandom aberrations in clear-cell sarcoma. *Cancer Genet Cytogenet* **64**: 107–110

Speleman F, Colpaert C, Goovaerts G, Leroy JG and Van Marck E (1992) Malignant melanoma of soft parts. Further cytogenetic characterization. *Cancer Genet Cytogenet* **58**: 176–179

Speleman F, Delattre O, Peter M, Hauben E, Van Roy N and Van Marck E (1997) Malignant melanoma of the soft parts (clear cell sarcoma): confirmation of EWS and ATF-1 gene fusion caused by t(12;22) translocation. *Mod Pathol* **10**(5): 496–499

Stenman G, Kindblom L-G and Angervall L (1992) Reciprocal translocation t(12;22)(q13;q13) in clear-cell sarcoma of tendons and aponeuroses. *Genes Chrom Cancer* **4**: 122–127

Travis JA and Bridge JA (1992) Significance of both numerical and structural chromosomal abnormalities in clear cell sarcoma. *Cancer Genet Cytogenet* **64**: 104–106

Vaandrager JW, Schuuring E, Zwikstra E, De Boer CJ, Kleiverda KK, Van Krieken JHJM, Kluin-Nelemans HC, Van Ommen GJB, Raap AK and Kluin PM (1996) Direct visualization of dispersed 11q13 chromosomal translocations in mantle cell lymphoma by multicolor DNA fiber fluorescence in situ hybridization. *Blood* **88**: 1177–1182

Zucman J, Delattre O, Desmaze C, Epstein AL, Stenman G, Speleman F, Fletcher CDM, Aurias A and Thomas G (1993) EWS and ATF-1 gene fusion induced by t(12;22) translocation in malignant melanoma of soft parts. *Nature Genet* **4**: 341–345

© 2001 Cancer Research Campaign
